# Supplementary material for: A digital single-molecule nanopillar SERS platform for predicting and monitoring immune toxicities in immunotherapy
Source: Nat Commun. 2021 Feb 17;12:1087. doi: 10.1038/s41467-021-21431-w (PMC7889912; doi:10.1038/s41467-021-21431-w)
Supplement: Supplementary file 1 — Supplementary Information [file 41467_2021_21431_MOESM1_ESM.pdf]

Supplementary Information

A digital single-molecule nanopillar SERS platform for predicting and monitoring immune toxicities in immunotherapy

Junrong Li[1], Alain Wuethrich[1]*, Abu A. I. Sina[1], Han-Hao Cheng[2], Yuling Wang[3]*, Andreas Behren[4,5], Paul N. Mainwaring[1] and Matt Trau[1,6]*

[1]Centre for Personalised Nanomedicine, Australian Institute for Bioengineering and Nanotechnology (AIBN), The University of Queensland, Brisbane, QLD 4072, Australia.

[2]Centre for Microscopy and Microanalysis, The University of Queensland, Brisbane, QLD 4072, Australia.

[3]Department of Molecular Sciences, ARC Centre of Excellence for Nanoscale BioPhotonics, Faculty of Science and Engineering, Macquarie University, Sydney, NSW 2109, Australia.

[4]Oliva Newton-John Cancer Research Institute, School of Cancer Medicine, La Trobe University, Heidelberg, VIC 3084, Australia.

[5]Department of Medicine, University of Melbourne, Heidelberg, VIC 3084, Australia.

[6]School of Chemistry and Molecular Biosciences, The University of Queensland, Brisbane, QLD 4072, Australia.

*Email: a.wuethrich@uq.edu.au; yuling.wang@mq.edu.au; m.trau@uq.edu.au.

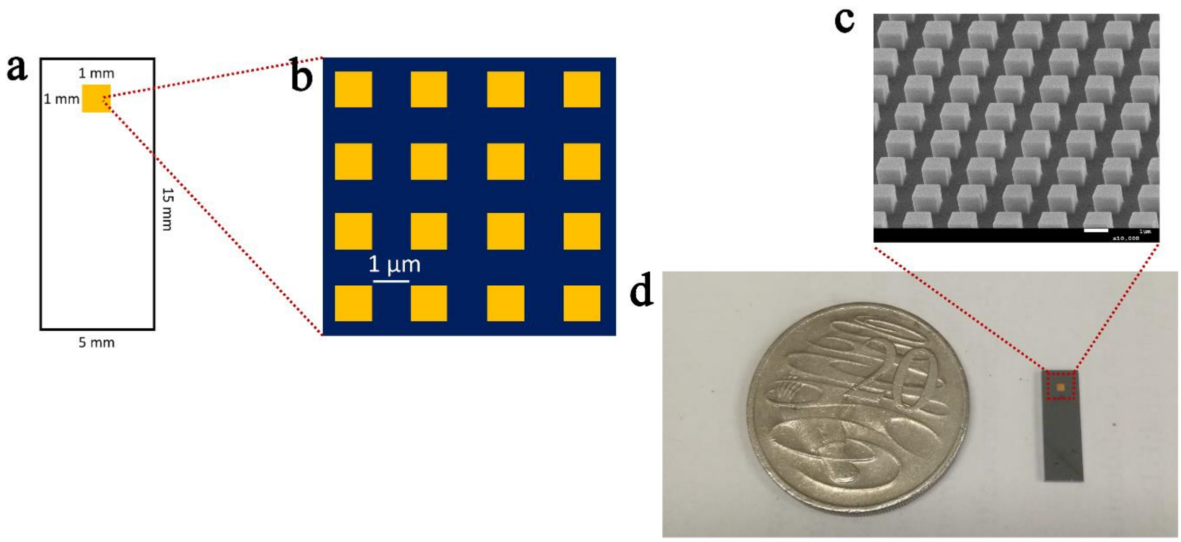

Supplementary Figure 1. Pillar array chip for digital nanopillar SERS assay. (a) Schematic of the pillar array for detecting single cytokine molecules, (b) inset shows pillar arrangement, (c) SEM image (top) of a small section of pillar array (scale bare = 1 µm), and (d) the photograph of pillar array chip (bottom).

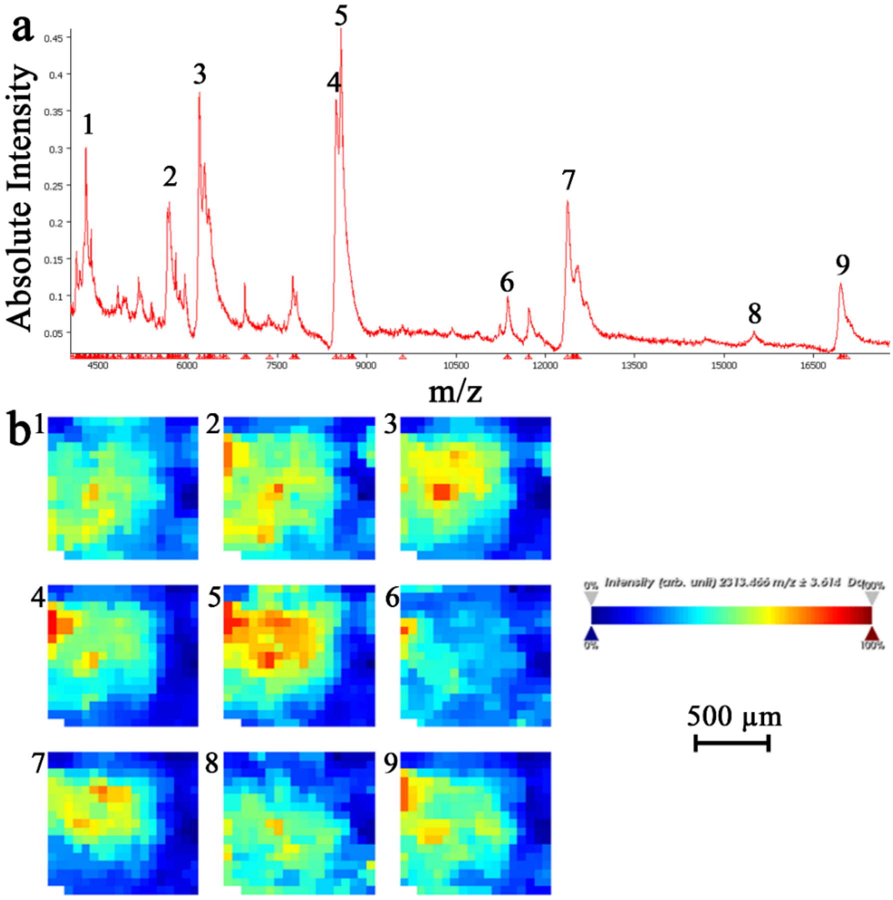

Supplementary Figure 2. Characterisation of antibody conjugation on the pillar array. (a) Averaged MALDI-TOF spectrum and (b) mass spectrometric mapping of pillar array that shows the distribution of antibody fragments for selected m/z of : (1) 4284, (2) 5649, (3) 6169, (4) 8482, (5) 8565, (6) 11750, (7) 12398, (8) 15497, and (9) 16979.

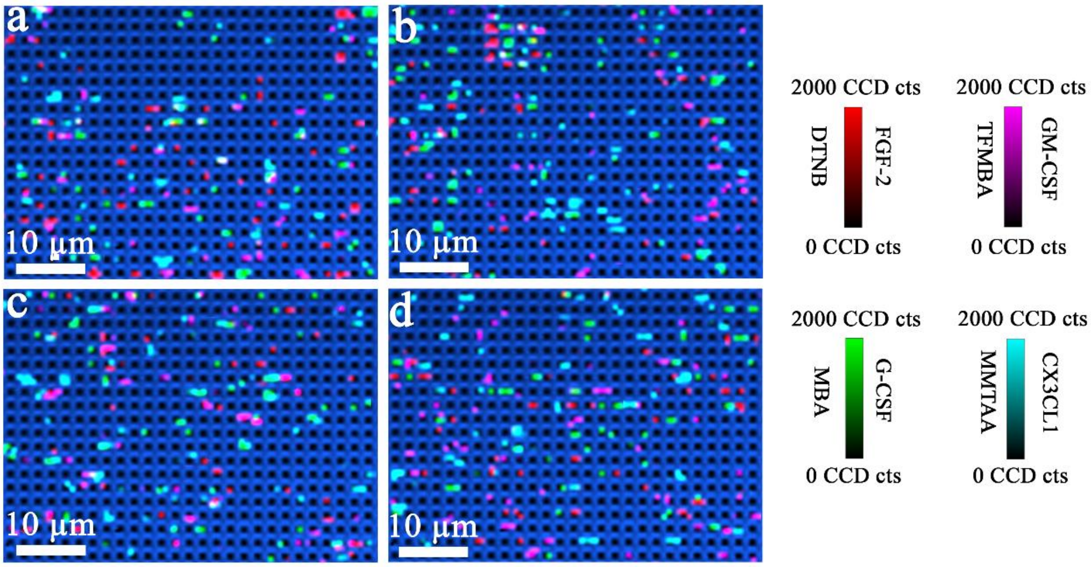

Supplementary Figure 3. Representative SERS mapping images obtained from the analysis of an equimolar cytokine mixture (1031 aM). The signal distribution of the SERS nanotags indicates a required conjugation and distribution of anti-fibroblast growth factor 2 (FGF-2), anti-granulocyte colony-stimulating factor (GM-CSF), anti-granulocyte colony-stimulating factor (G-CSF), and anti-fractalkine (CX3CL1) antibodies on the pillar array. Colour scale bars indicate Raman intensities from 5,5-dithiobis (2-nitrobenzoic acid) (DTNB), 4-mercaptobenzoic acid (MBA), 2,3,5,6-tetrafluoro-4-mercaptobenzoic acid (TFMBA), or 2-mercapto-4-methyl-5-thiazoleacetic acid (MMTAA). Data shown from one independent experiment.

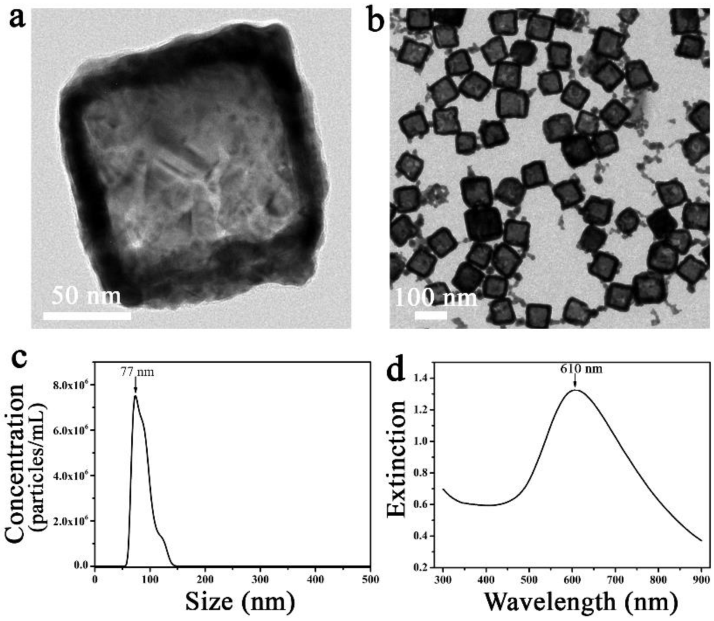

Supplementary Figure 4. Nanobox morphological and optical characterisation. (a, b) TEM images of nanoboxes; (c) nanobox size distribution; and (d) UV-vis extinction spectrum of nanoboxes. Data from one independent experiment. Source data are provided in the Source Data file.

Supplementary Table 1. Assignment of SERS peaks from four Raman reporters enhanced by nanoboxes.

Raman reporter	Raman peak (cm^{-1})	Assignment
DTNB	1060	Succinimidyl N-C-O stretching and aromatic ring vibration[1]
DTNB	1330	Symmetric NO_2 stretching[1]
DTNB	1556	Aromatic ring vibration[1]
MBA	1080	Aromatic ring vibration[2]
MBA	1580	Aromatic ring vibration[2]
TFMBA	1380	CH_2 deformation[3]
TFMBA	1631	NH_2 deformation[3]
MMTAA	1288	CH in-plane bending[4]

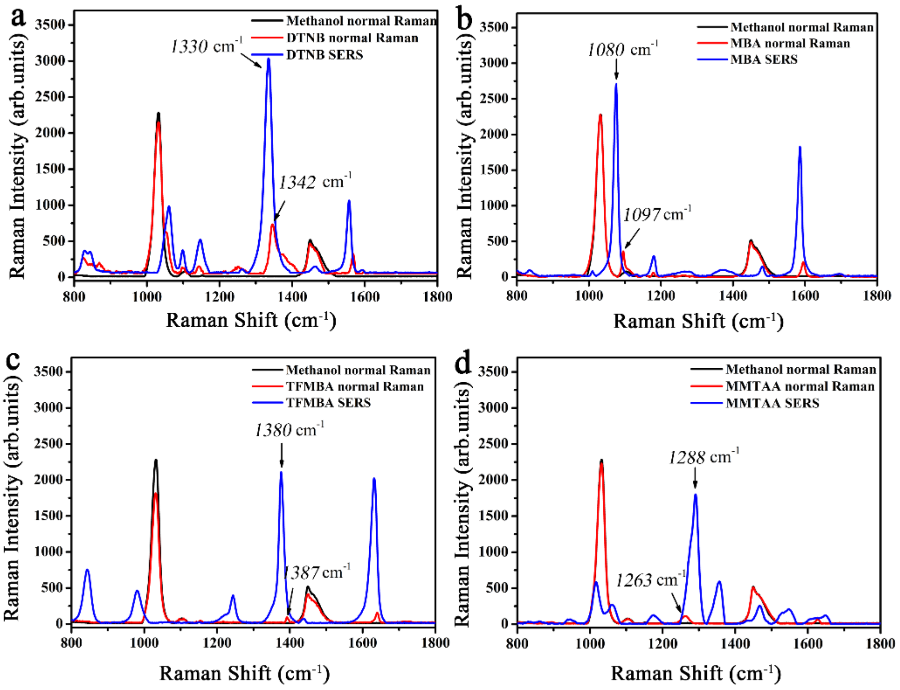

Supplementary Figure 5. Evaluating EFs of four Raman reporters on the nanoboxes. Surface-enhanced Raman scattering (SERS) and Raman spectra of (a) 5,5-dithiobis (2-nitrobenzoic acid) (DTNB), (b) 4-mercaptobenzoic acid (MBA), (c) 2,3,5,6-tetrafluoro-4-mercaptobenzoic acid (TFMBA), and (d) 2-mercapto-4-methyl-5-thiazoleacetic acid (MMTAA). The concentrations of Raman reporter in SERS and normal Raman measurement was 0.051µM and 100 mM, respectively. Each spectrum represents the average measurement from 10 Raman acquisition. Source data are provided in the Source Data file.

EF was calculated by the following formula:

$$EF = (I_{SERS}/N_{SERS})/(I_{RS}/N_{RS})$$

where I_{SERS} and N_{SERS} are Raman intensity and the number of molecules in SERS measurement, respectively. I_{RS} and N_{RS} are Raman intensity and the number of molecules in normal Raman measurement, respectively.

To allow a robust calculation of EF, liquid samples with Raman reporters on nanobox surfaces or dissolved in methanol were tested in a cuvette. As these four Raman reporters all utilised thiol group to attach the nanobox surfaces, we assumed they formed a self-assembly monolayer with the same coverage of 0.5 nmol/cm^2 based on the previous report (Anal. Chem., 2005, 77, 3261-3266). The nanoboxes had the concentration of 2.95×10^8 particles/mL and the mode size of 77 nm according to NTA characterisation. The concentration of Raman molecules on the nanobox surfaces was thus determined to be 0.051 µM. The normal Raman measurement was conducted with 100 mM of each Raman reporter in methanol. Supplementary Figure 4 shows the representative SERS and normal Raman spectra of each Raman reporter measurement. It is worth noting that SERS spectra shifted in wavenumber compared to the normal Raman spectra probably due to the strong interactions of molecule with gold-silver surfaces, which was similarly observed in literature (e.g., Anal. Chem., 2003, 75, 5936-5943; Molecules, 2008, 13, 2608-2627). Based on the labelled characteristic peaks in Supplementary Figure 4, the calculated EF of DTNB, MBA, TFMBA, and MMTAA was 8.14×10^6, 1.46×10^7, 4.01×10^7, and 3.26×10^7, respectively.

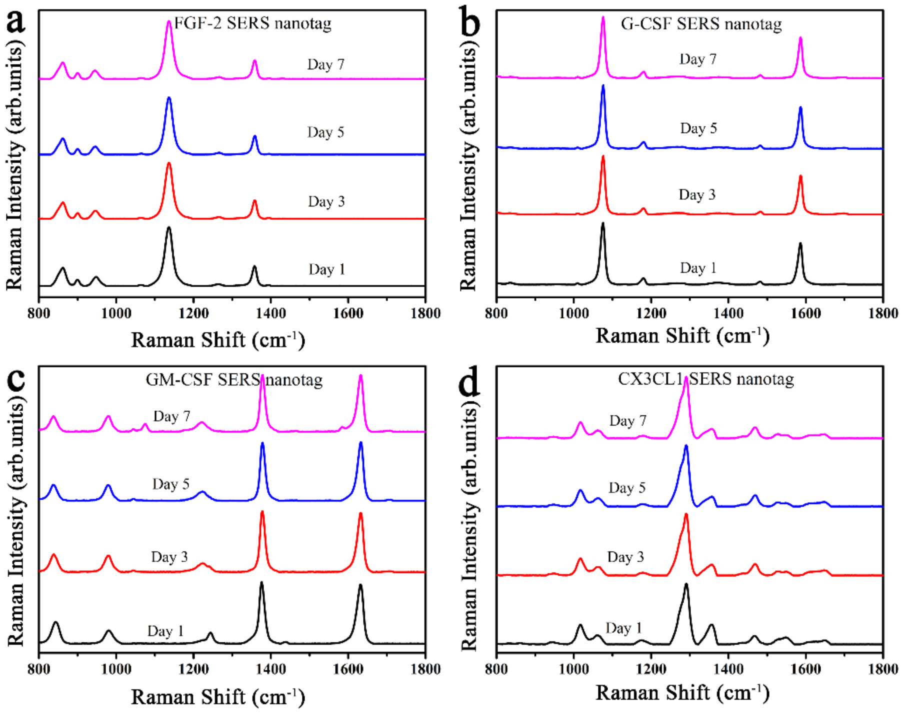

Supplementary Figure 6. Monitoring the signal stability of the surface-enhaced Raman scattering (SERS) nanotags over 7 days. SERS spectra of (a) fibroblast growth factor 2 (FGF-2), (b) granulocyte colony-stimulating factor (GM-CSF), (c) granulocyte colony-stimulating factor (G-CSF), and (d) fractalkine (CX3CL1) nanotags on day 1, day 3, day 5, and day 7. Each spectrum represents the average measurement from 10 Raman acquisition. Source data are provided in the Source Data file.

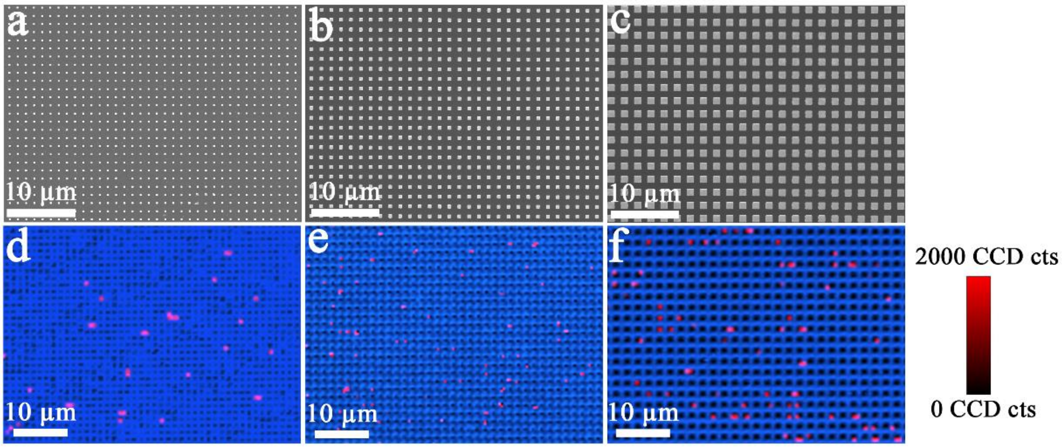

Supplementary Figure 7. Optimisation of pillar sizes. SEM images of pillar array of (a) 250 nm, (b) 500 nm, (c) 1000 nm; SERS images for FGF-2 counting on pillar array of (d) 250 nm, (e) 500 nm, (f) 1000 nm. The median (interquartile range) of active pillars per scanning image for 250 nm, 500 nm, and 1000 nm pillar array was 30 (27.5-32.5), 74 (73-81), and 68 (62-76), respectively. Data shown from one independent experiment.

Supplementary Table 2. Optimisation of target capture efficiency using pillar array with three sizes.

	250 nm active pillar%		500 nm active pillar/%		1000 nm active pillar/%	
	Theoretical	Experimental	Theoretical	Experimental	Theoretical	Experimental
1031aM	10.00	1.90	10.00	5.90	10.00	10.05
260 aM	2.50	0.37	2.50	1.00	2.50	2.08

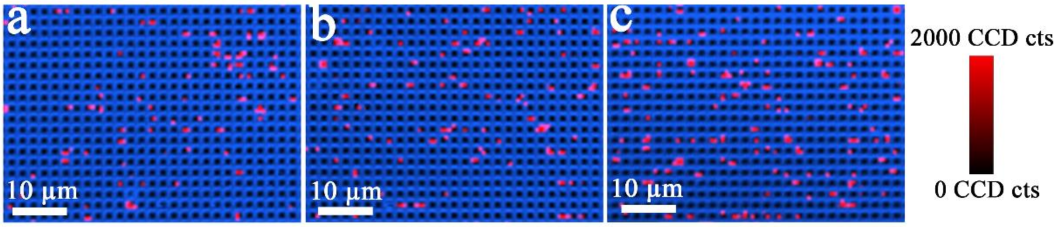

Supplementary Figure 8. Optimisation of incubation time. FGF-2 incubated with pillar array for (a) 30 min, (b) 60 min, and (c) 90 min. The median (interquartile range) of active pillars per scanning image for 30 min, 60 min, and 90 min incubation was 61 (60-77), 62 (61-68), 70 (60-70), respectively. Data shown from one independent experiment.

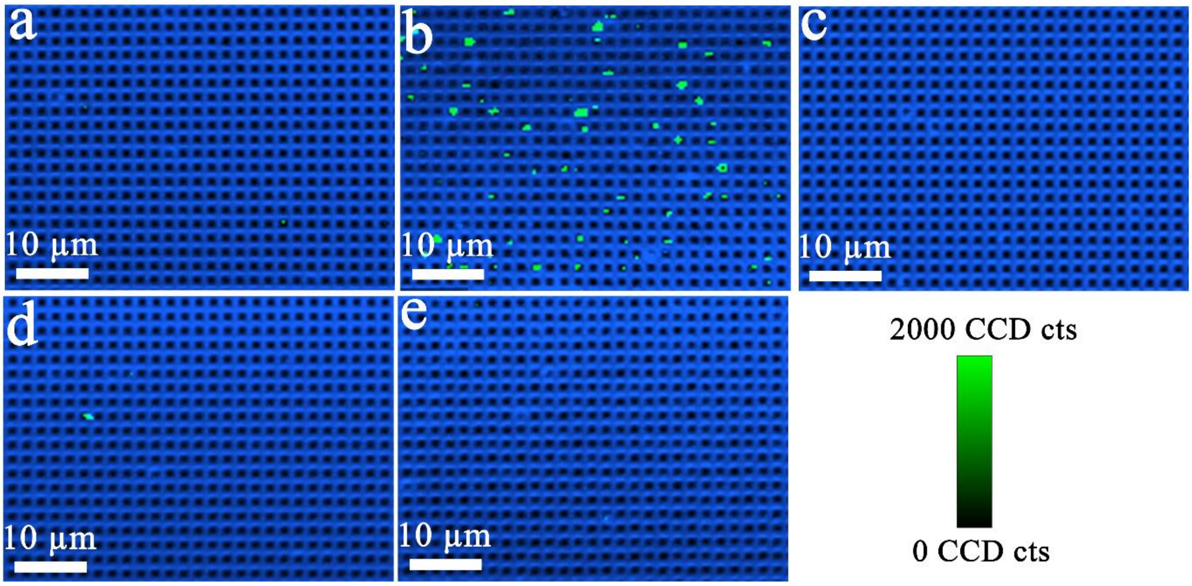

Supplementary Figure 9. Demonstration of G-CSF detection specificity. SERS images for G-CSF SERS nanotags in the presence of (a) FGF-2, (b) G-CSF, (c) GM-CSF, (d) CX3CL1, and (e) PBS. The median (interquartile range) of active pillars per scanning image for FGF-2, G-CSF, GM-CSF, CX3CL1 and PBS was 2 (1.5-5.5), 70 (58.5-77.5), 2 (0.5-2), 2 (1-2), and 2 (1.5-4), respectively. Data shown from one independent experiment.

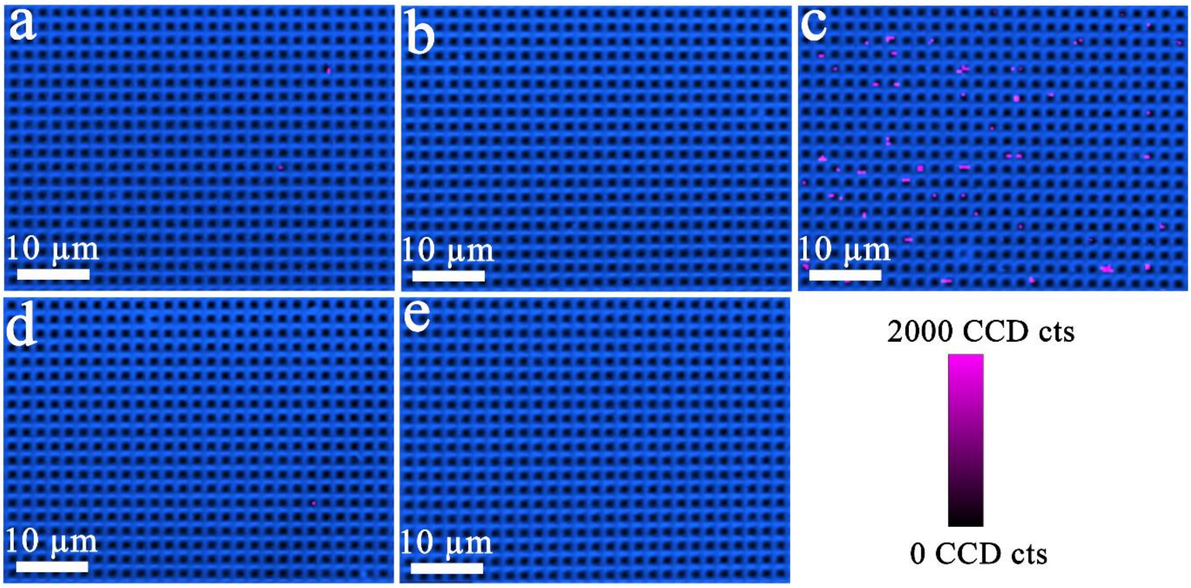

Supplementary Figure 10. Demonstration of GM-CSF detection specificity. SERS images for GM-CSF SERS nanotags in the presence of (a) FGF-2, (b) G-CSF, (c) GM-CSF, (d) CX3CL1, and (e) PBS. The median (interquartile range) of active pillars per scanning image for FGF-2, G-CSF, GM-CSF, CX3CL1 and PBS was 3.5 (2.75-4.25), 0 (0-1), 43 (38.75-51), 2 (1-2), and 0 (0-1), respectively. Data shown from one independent experiment.

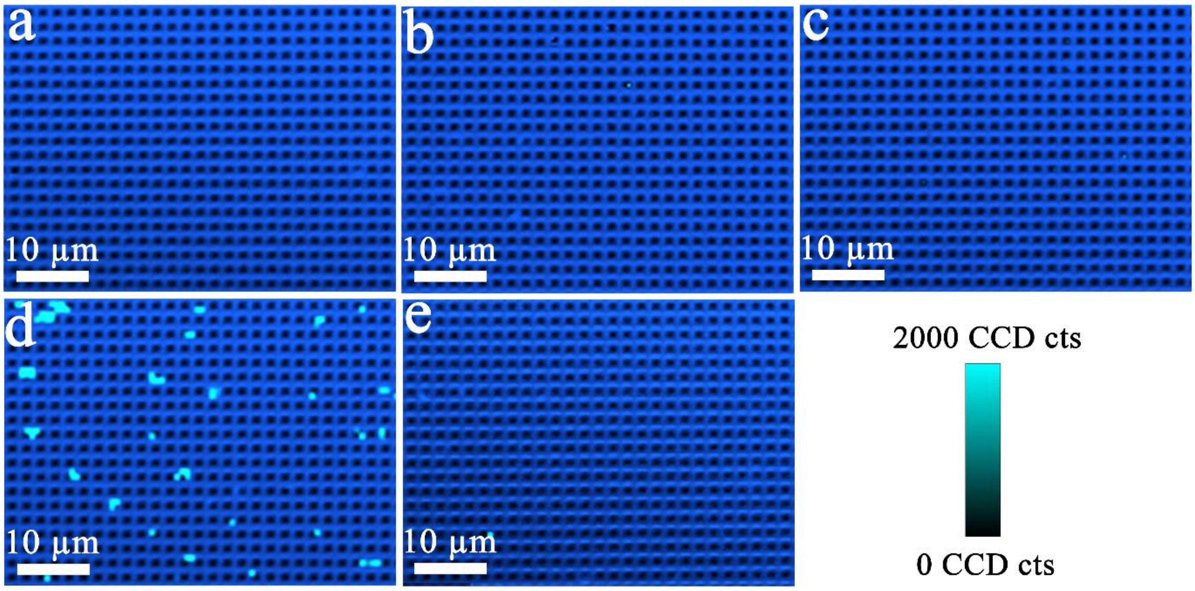

Supplementary Figure 11. Demonstration of CX3CL1 detection specificity. SERS images for CX3CL1 SERS nanotags in the presence of (a) FGF-2, (b) G-CSF, (c) GM-CSF, (d) CX3CL1, and (e) PBS. The median (interquartile range) of active pillars per scanning image for FGF-2, G-CSF, GM-CSF, CX3CL1 and PBS was 1 (0.5-1.5), 0 (0-2), 1 (0.5-2), 35 (33-36), and 1 (0.5-1.5), respectively. Data shown from one independent experiment.

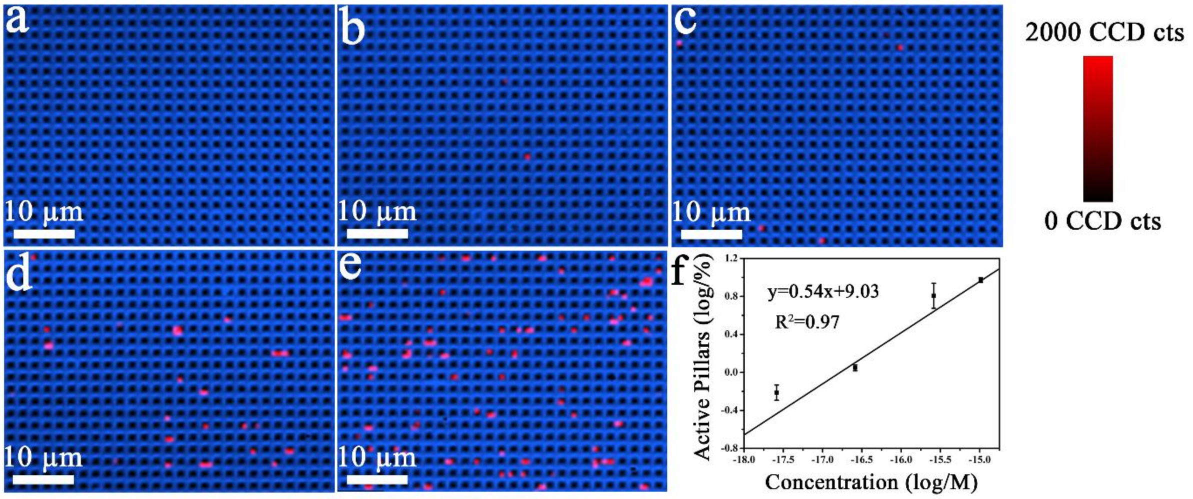

Supplementary Figure 12. Sensitivity for the detection of FGF-2. SERS images of FGF-2 with the concentration of (a) 0 aM, (b) 2.6 aM, (c) 26 aM, (d) 260 aM, (e) 1031 aM; (f) linear relationship curve. The median (quartile range) of active pillar number per scanning image for 0, 2.6 aM, 26 aM, 260 aM, and 1031 aM was 2 (1-4), 4 (2-6), 8 (5.75-9), 44 (34-55.5), 64 (60-74.25). The error bars represented the standard deviation from three independent technical measurements on three chips. The centre of the error bars represented the average value of the measurements. Source data are provided in the Source Data file.

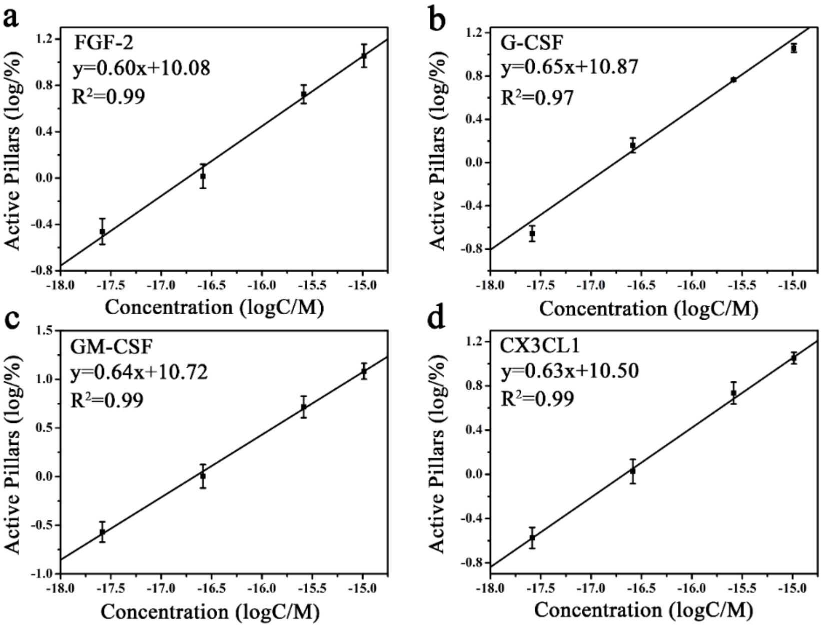

Supplementary Figure 13. Sensitivity for the simultaneous detection of four cytokine molecules. Linear relationship curve for the detection of (a) fibroblast growth factor 2 (FGF-2), (b) granulocyte colony-stimulating factor (GM-CSF), (c) granulocyte colony-stimulating factor (G-CSF), and (d) fractalkine (CX3CL1). The error bars represented the standard deviation from three independent technical measurements on three chips. The centre of the error bars represented the average value of the measurements. Source data are provided in the Source Data file.

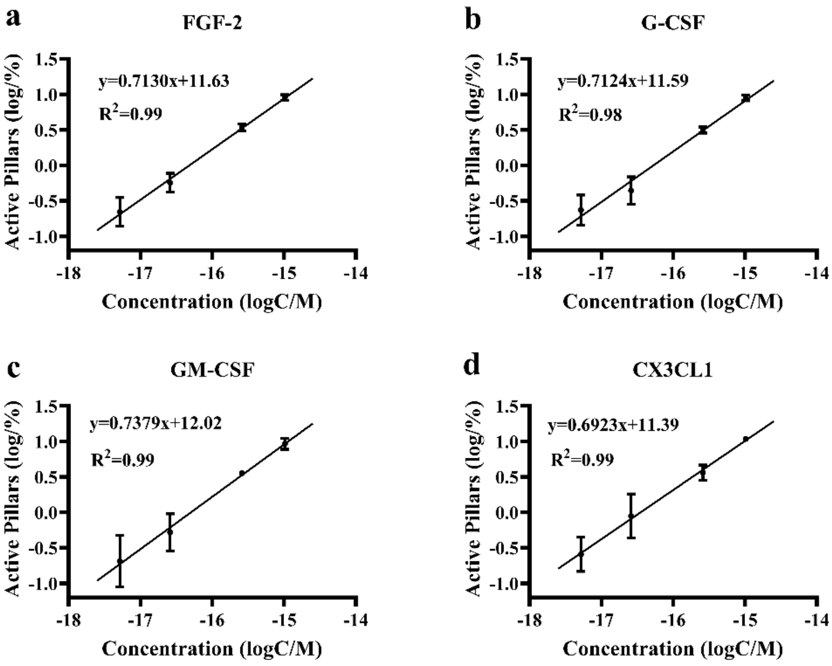

Supplementary Figure 14. Sensitivity for the simultaneous detection of four cytokine molecules in human serum. Linear relationship curve for the detection of (a) fibroblast growth factor 2 (FGF-2), (b) granulocyte colony-stimulating factor (GM-CSF), (c) granulocyte colony-stimulating factor (G-CSF), and (d) fractalkine (CX3CL1), respectively. The error bars represented the standard deviation from three independent technical measurements on three chips. The centre of the error bars represented the average value of the measurements. Source data are provided in the Source Data file.

Supplementary Table 3. Comparison of experimental and theoretical possibility of finding different molecule numbers on pillar array.

Molecule number	1	2	3	4
Experimental probability (%)	11.52±0.37	0.83±0.041	0.036±0.0073	0.0206±0.0073
Theoretical probability (%)	9.05	0.45	0.015	0.0004

The experimental data is based on three technical replicates on three chips with the data showing mean ± S.D.

To study the experimental Poisson distribution, we used the chips with four cytokines and SERS nanotags to have a total of 10% active pillars (i.e., each cytokine activated 2.5% pillars). The experimental probability of finding 1, 2, 3, and 4 molecules on a pillar was calculated by counting 1, 2, 3, and 4 types of specific Raman reporter peaks on a pillar in the SERS mapping images. The theoretical probability was determined with the formula $P(X=\mathbf{k}) = \frac{\lambda^k e^{-\lambda}}{k!}$, where k is the number of molecules observed on a single pillar (i.e., 1, 2, 3, 4), λ is expected molecule number on a single pillar (i.e., 0.1), e is Euler's number.

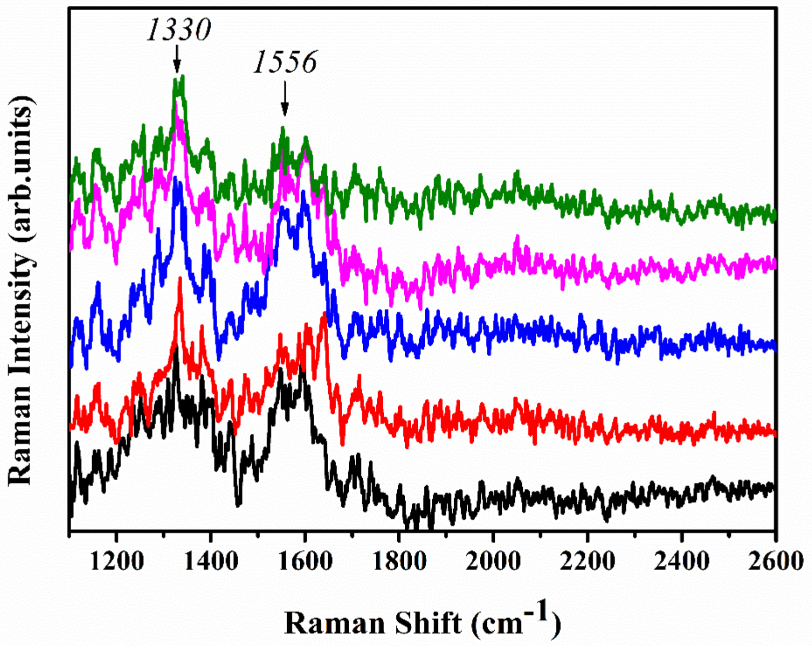

Supplementary Figure 15. Representative SERS spectra of FGF-2 SERS nanotags in detecting FGF-2 in human serum. The two labelled peaks are from DTNB reporters in FGF-2 SERS nanotags. Source data are provided in the Source Data file.

Supplementary Table 4. Detection of cytokines in healthy (blank) human serum on five independent chips.

Serum	Chip 1 (fM)	Chip 2 (fM)	Chip 3 (fM)	Chip 4 (fM)	Chip 5 (fM)	RSD (%)	p Value[a]
FGF-2	8.29	8.45	7.40	7.10	7.58	6.72	0.49
G-CSF	6.19	6.98	6.42	6.14	7.15	6.28	0.56
GM-CSF	8.01	8.57	8.16	7.66	7.94	3.69	0.95
CX3CL1	7.28	6.63	7.22	5.87	6.27	8.18	0.22

[a]Kruskal Wallis test followed by Dunn's test to correct multiple comparisons (two-sided). A nonparametric test to statistically determine the significant differences between two or more groups.

Supplementary Table 5. Detection of cytokines in human serum spiked with 1 fM standards on five independent chips.

Spiked Serum	Chip 1 (fM)	Chip 2 (fM)	Chip 3 (fM)	Chip 4 (fM)	Chip 5 (fM)	RSD (%)	p Value[a]
FGF-2	9.11	9.90	8.34	8.55	9.11	5.52	0.88
G-CSF	7.40	8.00	8.02	7.47	8.82	5.86	0.84
GM-CSF	8.81	9.45	9.00	8.79	9.22	2.54	0.95
CX3CL1	8.06	7.55	7.99	6.44	7.24	7.20	0.68

[a]Kruskal Wallis test followed by Dunn's test to correct multiple comparisons (two-sided). A nonparametric test to statistically determine the significant differences between two or more groups.

Supplementary Table 6. Recovery test of four cytokines in spiked human serum.

Cytokines	Added (fM)	Detected (fM)	Recovery (%)	RSD (%)
FGF-2	1.00	1.24	124.00	21.80
G-CSF	1.00	1.37	137.00	16.19
GM-CSF	1.00	0.99	99.00	17.29
CX3CL1	1.00	0.80	80.00	16.02

Supplementary Table 7. Comparison of digital nanopillar SERS and ELISA on three simulated clinical samples. The FGF-2, G-CSF, GM-CSF, and CX3CL1 concentrations were in sample 1 (a) 3.64 pM, 3.19 pM, 4.29 pM, and 28.57 pM; sample 2 (b) 14.56 pM, 12.76 pM, 17.16 pM, and 114.28 pM; and sample 3 (c) 58.24 pM, 51.04 pM, 68.64 pM, and 457.12 pM.

	Sample 1 (pM)		Sample 2 (pM)		Sample 3 (pM)	
	SERS	ELISA	SERS	ELISA	SERS	ELISA
FGF-2	2.43±1.17	1.31±0.13	11.06±4.35	9.00±0.87	45.85±6.03	38.47±2.57
G-CSF	2.56±1.02	3.30±0.24	11.63±1.56	16.59±1.14	44.53±11.33	53.80±3.25
GM-CSF	5.27±0.89	4.84±0.15	18.75±4.29	19.53±0.46	78.42±25.41	54.24±4.02
CX3CL1	24.36±1.22	24.92±3.98	84.03±28.27	93.48±19.84	440.59±96.75	565.75±168.68

Supplementary Table 8. Detection of cytokines in human serum and spiked serum samples with digital nanopillar SERS platform and ELISA assay. The spiked FGF-2, G-CSF, GM-CSF, and CX3CL1 concentrations in human serum were 14.56 pM, 12.76 pM, 17.16 pM, and 114.28 pM, respectively.

Cytokine	Serum		Spiked serum	
	SERS (fM)	ELISA	SERS (pM)	ELISA (pM)
FGF-2	8.05±0.46	N/A	19.49±1.51	20.20±0.50
G-CSF	6.53±0.33	N/A	11.26±2.12	10.77±0.53
GM-CSF	8.25±0.23	N/A	16.06±1.88	18.76±0.06
CX3CL1	7.05±0.29	N/A	169.22±40.77	171.02±16.58

N/A: not applicable

Supplementary Table 9. Concentration of 4 cytokines in the serum of 10 healthy people as determined by digital nanopillar SERS platform.

	FGF-2 (fM)	G-CSF (fM)	GM-CSF (fM)	CX3CL1 (fM)
H1	8.05±0.46	6.53±0.33	8.25±0.23	7.05±0.29
H2	5.77±1.04	1.82±0.55	3.29±0.57	3.90±0.96
H3	1.46±0.42	0.35±0.27	0.75±0.46	1.19±0.81
H4	7.40±1.36	2.15±1.09	5.87±1.09	4.48±1.38
H5	2.93±0.89	1.13±0.36	2.04±1.18	1.68±0.96
H6	2.95±0.69	1.73±0.25	2.24±0.86	1.19±0.58
H7	4.89±1.06	1.00±0.44	3.53±0.55	3.04±0.92
H8	8.59±1.05	1.57±0.67	4.69±0.40	3.16±0.32
H9	5.94±0.99	4.85±1.29	6.71±0.99	1.22±0.07
H10	1.46±0.54	1.94±0.95	1.66±0.72	1.23±0.57

The experimental data is based on three technical replicates on three chips with the data showing mean ± S.D..

Supplementary Table 10. Demographic information for melanoma patients

Patient	Age	Gender	Stage	Sites of metastases	Clinical trial	Treatment agent	Serum/Plasma dates	Response (RECIST or iRC)	irAEs ?	irAE onset	irAE grade	irAE type
1	43	M	IV	subcutaneous, LN	LUD2012-003	BCG, Ipilimumab	1A: C1D7 (9/10/2014)	SD	Yes			
							1B: C2D1 (23/10/2014)					
							1C: C3D1 (13/11/2014)			26/11/2014	4	colitis
2	68	M	IV	heart, brain	NA	Ipilimumab	2A:C1D1 (21/05/2015)	SD	Yes	1/06/2015	3	skin
					NA	Nivolumab	2B: D70 (16/09/2015)					
3	75	M	IV	unknown	MK-3475-029	Pembrolizumab + Ipilimumab	3A: C1D1 (11/09/2015)	CR	Yes	29/09/2015	3	skin
							3B: C1D22 (1/10/2015)					
							3C: C2D1 (23/10/2015)					
4	47	M	IV	unknown	MK-3475-029	Pembrolizumab + Ipilimumab	4A: C1D1 (21/05/2015)	CR	Yes	26/06/2015	4	pancreas
							4B: C2D1 (24/07/2015)					
5	71	M	IV	lung	MK-3475-029	Ipilimumab	5A: C1D1 (28/07/2015)	CR	Yes			
							5B: C1D22 (19/08/2015)			26/08/2015	2	skin
							5C: C2D1 (10/09/2015)			1/10/2015	3	lung
6	63	M	IV	lung, LN, liver	MK-3475-029	Pembrolizumab + Ipilimumab	6A: C1D1 (4/06/2015)	PR	Yes			
							6B: C1D22 (25/06/2015)			28/06/2015	2	skin
							6C: C2D1 (16/07/2015)					
7	74	M	IV	lung	MK-3475-029	Pembrolizumab + Ipilimumab	7A: C1D1 (2/09/2015)	CR	Yes			
							7B: C1D22 (24/09/2015)					
							7C: C2D1 (15/10/2015)			17/12/2015	2	arthritis
8	60	M	IV	bone, LN	MK-3475-029	Pembrolizumab + Ipilimumab	8A: C1D1 (24/06/2015)	PR	No			
							8B: C1D22 (17/07/2015)					
							8C: C2D1 (7/08/2015)					
9	57	M	unknown	unknown	MK-3475-029	Ipilimumab	9A: C1D1 (14/05/2015)	PD	Yes	5/06/2019	1	skin
							9B: C3D1 (6/08/2015)					
10	49	M	IV	lung	unknown	Ipilimumab	10A: C1D1 (31/08/2011)	PD	Yes	21/09/2011	1	skin
							10B: D84 (12/10/2011)					

LN-lymph node
MK-3475-029 -clinical trial Pembrolizumab + Ipilimumab Q3W
LUD2012-003-BCG followed by Ipilimumab, cycle refers to Ipilimumab
C-cycle
D-day
SD-stable disease
CR-complete response
PR-partial response
PD-progressive disease

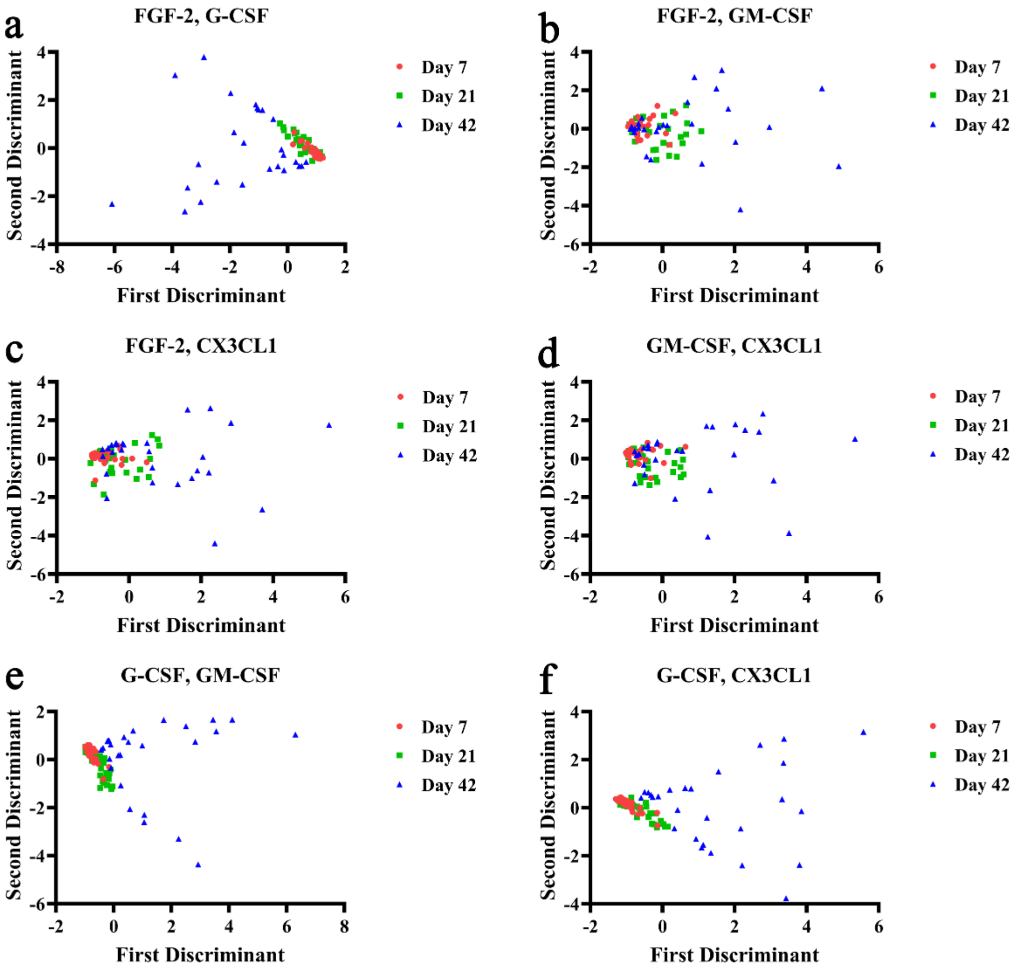

Supplementary Figure 16. LDA of patient 1 who developed severe irAEs (grade 4) with the use of two cytokines. Cytokines: fibroblast growth factor 2 (FGF-2), granulocyte colony-stimulating factor (GM-CSF), granulocyte colony-stimulating factor (G-CSF), and fractalkine (CX3CL1). Source data are provided in the Source Data file.

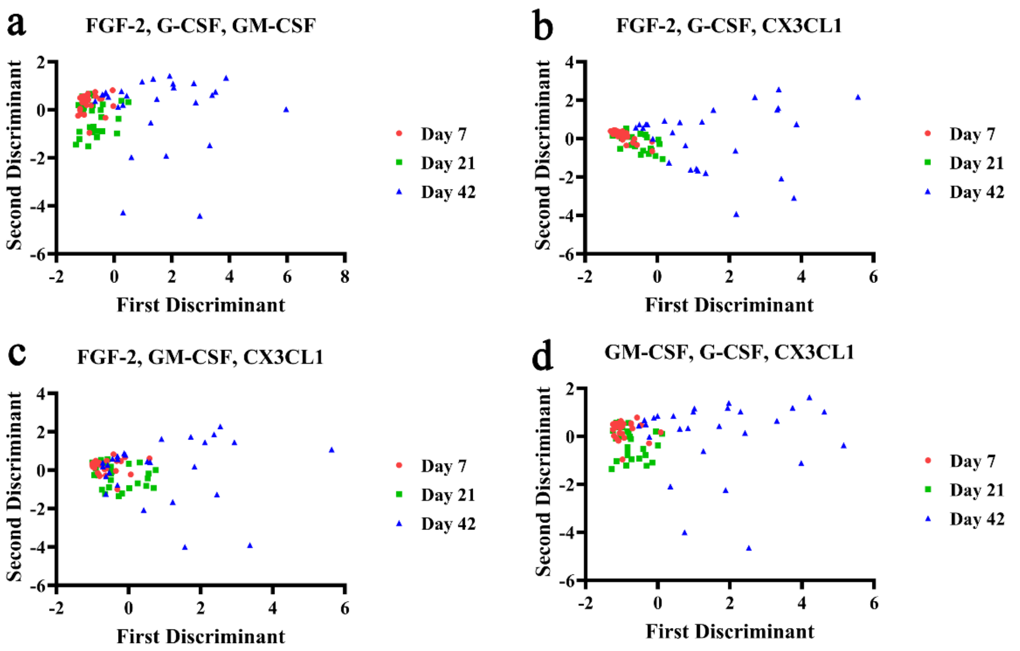

Supplementary Figure 17. LDA of patient 1 who developed severe irAEs (grade 4) with the use of three cytokines. Cytokines: fibroblast growth factor 2 (FGF-2), granulocyte colony-stimulating factor (GM-CSF), granulocyte colony-stimulating factor (G-CSF), and fractalkine (CX3CL1). Source data are provided in the Source Data file.

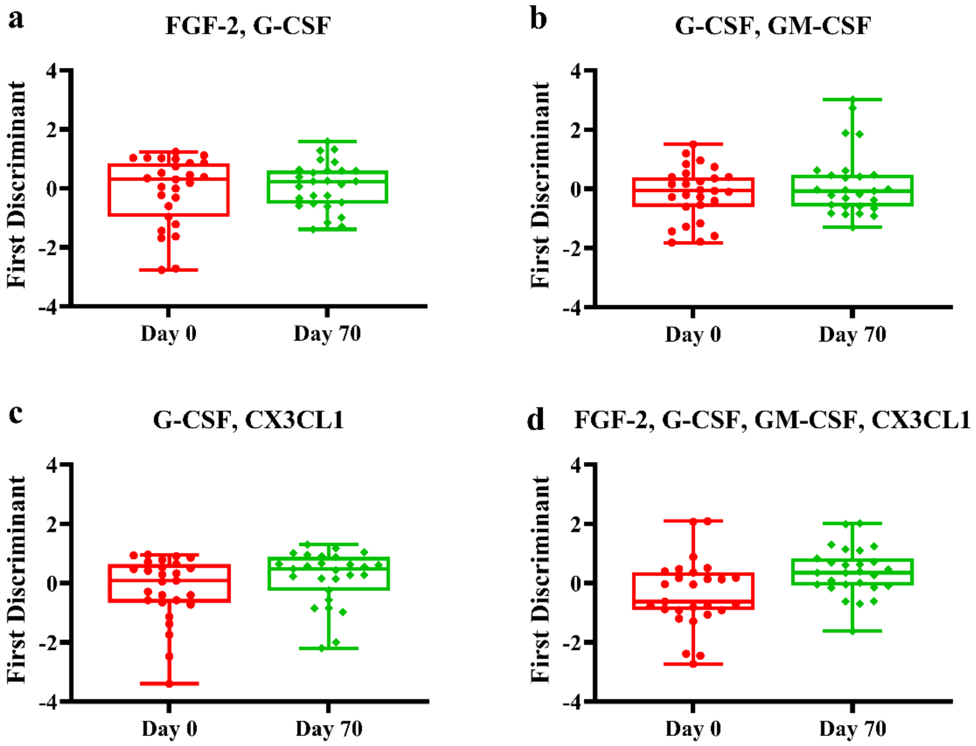

Supplementary Figure 18. LDA of patient 2 who developed severe irAEs (grade 3) with the use of different combinations of cytokines. The middle lines of the boxes represent the median (50th percentile) and the terminal lines of the boxes represent the 25th and 75th percentile. Cytokines: fibroblast growth factor 2 (FGF-2), granulocyte colony-stimulating factor (GM-CSF), granulocyte colony-stimulating factor (G-CSF), and fractalkine (CX3CL1). Source data are provided in the Source Data file.

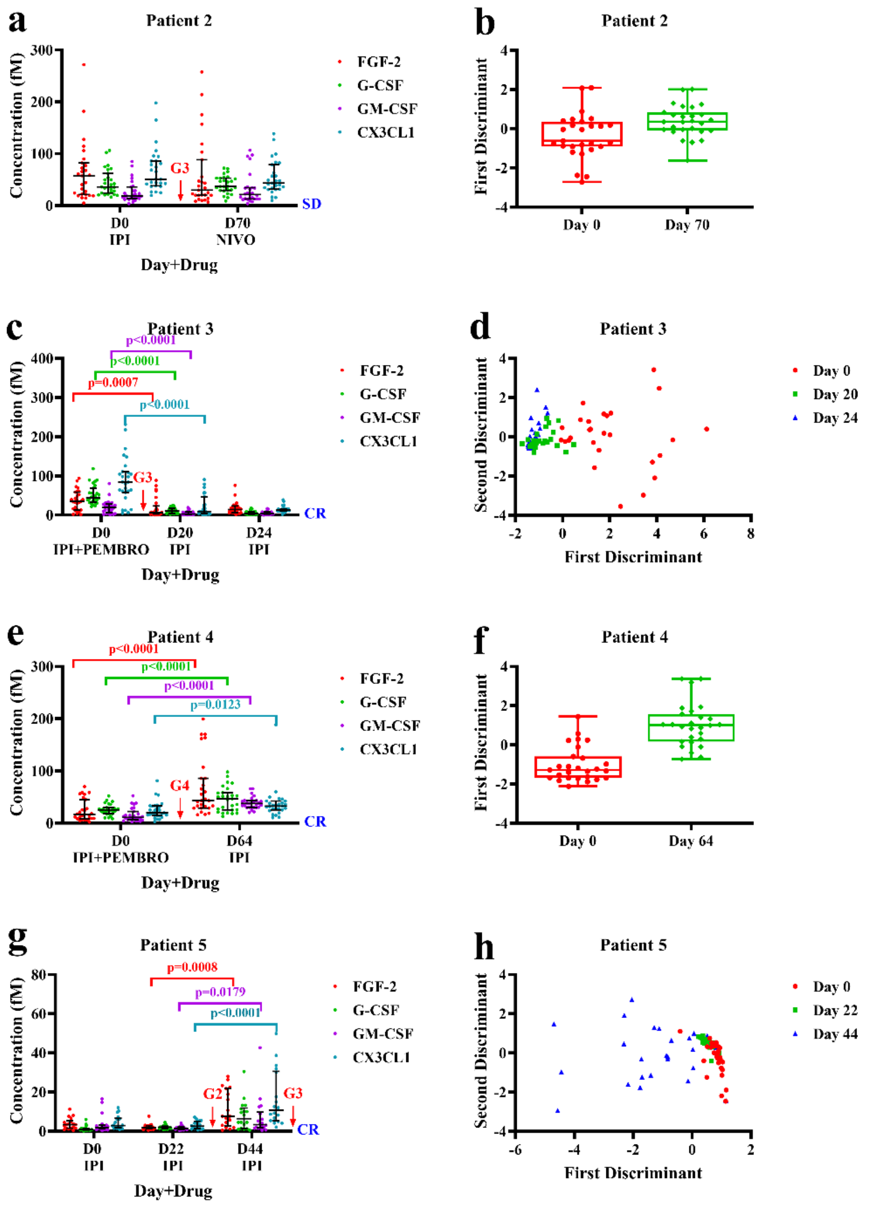

Supplementary Figure 19. Digital nanopillar SERS assay for monitoring melanoma patients who developed irAEs during immune checkpoint therapy. The average cytokine concentration graph (a, c, e, and g), the two shorter horizontal lines denote the interquartile ranges and the longer horizontal lines in between denote the median ; and corresponding LDA (b, d, f, and h), respectively. LDA discriminates the groups or clusters using the linear combination of four cytokine biomarkers. The middle lines of the boxes represent the median (50th percentile) and the terminal lines of the boxes represent the 25th and 75th percentile. IPI = ipilimumab, PEMBRO = pembrolizumab; G3=grade 3, G2=grade 2; SD=stable disease, PR=partial response.The data represented three technical replicates by testing on three chips with each chip for counting on nine images. Cytokines: fibroblast growth factor 2 (FGF-2), granulocyte colony-stimulating factor (GM-CSF), granulocyte colony-stimulating factor (G-CSF), and fractalkine (CX3CL1). Statistical analysis was conducted with Kruskal Wallis test among three groups followed by Dunn's test to correct multiple comparisons or Mann-Whitney test between two groups (two-sided). Source data are provided in the Source Data file.

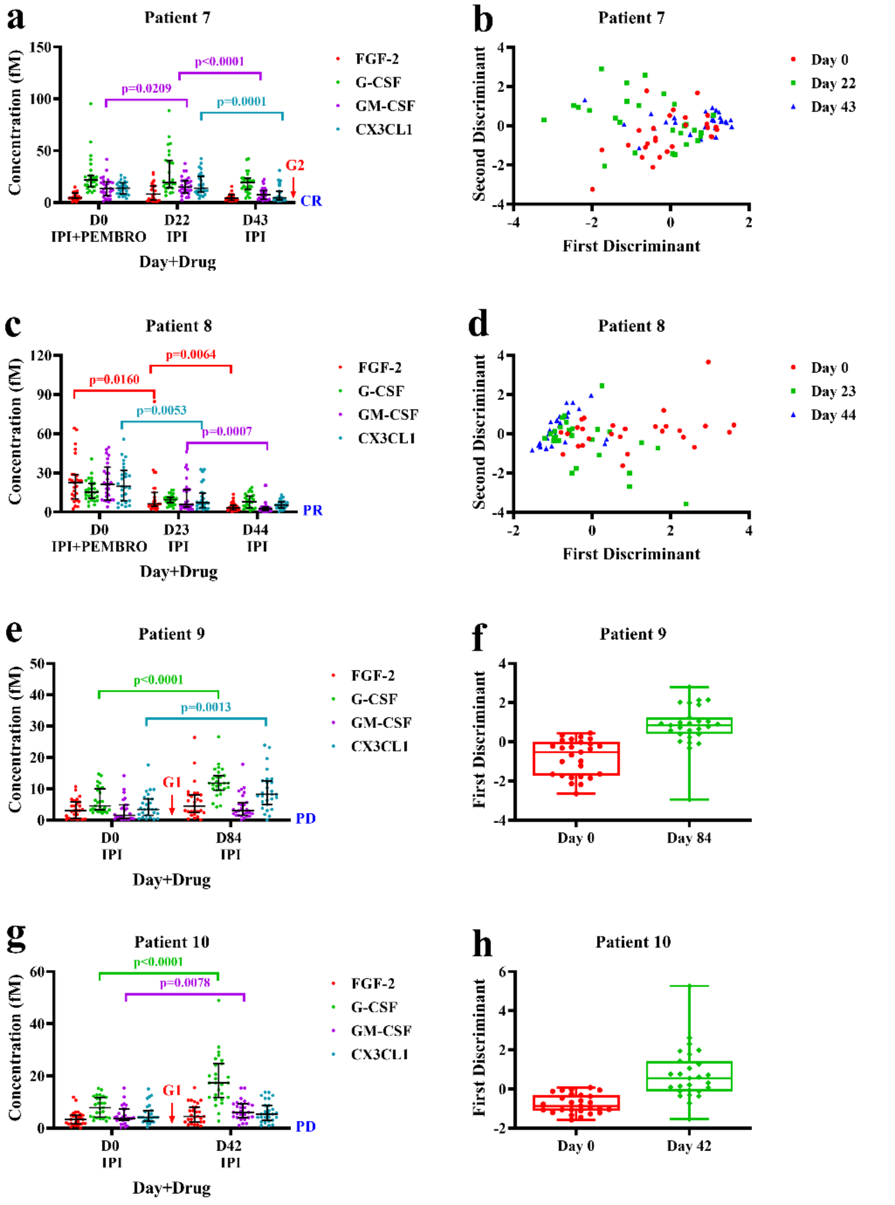

.

Supplementary Figure 20. Digital nanopillar SERS assay for monitoring melanoma patients who had not developed severe irAEs during immune checkpoint therapy. The average cytokine concentration graph (a, c, e, and g), the two shorter horizontal lines denote the interquartile ranges and the longer horizontal lines in between denote the median; and corresponding LDA (b, d, f, and h), respectively. LDA discriminates the groups or clusters using the linear combination of four cytokine biomarkers. The middle lines of the boxes represent the median (50th percentile) and the terminal lines of the boxes represent the 25th and 75th percentile. IPI = ipilimumab, PEMBRO = pembrolizumab; G3=grade 3, G2=grade 2; SD=stable disease, PR=partial response. The data represented three technical replicates obtained from three chips and nine images were acquired from each chipfor analysis. Cytokines: fibroblast growth factor 2 (FGF-2), granulocyte colony-stimulating factor (GM-CSF), granulocyte colony-stimulating factor (G-CSF), and fractalkine (CX3CL1). Statistical analysis was conducted with Kruskal Wallis test among three groups followed by Dunn’s test to correct multiple comparisons or Mann-Whitney test between two groups (two-sided). Source data are provided in the Source Data file.

Supplementary References:

1. Grubisha, D. S., Lipert, R. J., Park, H. Y., Driskell, J. & Porter, M. D. Femtomolar detection of prostate-specific antigen: An immunoassay based on surface-enhanced Raman scattering and immunogold labels. *Anal. Chem.* **75**, 5936–5943 (2003).
2. Zhu, S. *et al.* Realization of high sensitive SERS substrates with one-pot fabrication of Ag-Fe3O4 nanocomposites. *J. Colloid Interface Sci.* **438**, 116–121 (2015).
3. Arjunan, V., Devi, L. & Mohan, S. Conformational analysis, spectroscopic, structure–activity relations and quantum chemical simulation studies of 4–(trifluoromethyl)benzylamine. *J. Mol. Struct.* **1159**, 103–117 (2018).
4. Zhu, D. *et al.* Investigating the intracellular behaviors of liposomal nanohybrids via SERS: Insights into the influence of metal nanoparticles. *Theranostics* **8**, 941–954 (2018).
